# Vigi4Med Scraper: A Framework for Web Forum Structured Data Extraction and Semantic Representation

**DOI:** 10.1371/journal.pone.0169658

**Published:** 2017-01-25

**Authors:** Bissan Audeh, Michel Beigbeder, Antoine Zimmermann, Philippe Jaillon, Cédric Bousquet

**Affiliations:** 1 University of Lyon, MINES Saint-Étienne, CNRS, Hubert Curien Laboratory, UMR 5516, Saint-Étienne, France; 2 Ecole Nationale Supérieure des Mines de Saint-Étienne, Saint-Étienne, France; 3 INSERM, U1142, LIMICS, Paris, France; University of Texas at San Antonio, UNITED STATES

## Abstract

The extraction of information from social media is an essential yet complicated step for data analysis in multiple domains. In this paper, we present Vigi4Med Scraper, a generic open source framework for extracting structured data from web forums. Our framework is highly configurable; using a configuration file, the user can freely choose the data to extract from any web forum. The extracted data are anonymized and represented in a semantic structure using Resource Description Framework (RDF) graphs. This representation enables efficient manipulation by data analysis algorithms and allows the collected data to be directly linked to any existing semantic resource. To avoid server overload, an integrated proxy with caching functionality imposes a minimal delay between sequential requests. Vigi4Med Scraper represents the first step of Vigi4Med, a project to detect adverse drug reactions (ADRs) from social networks founded by the French drug safety agency Agence Nationale de Sécurité du Médicament (ANSM). Vigi4Med Scraper has successfully extracted greater than 200 gigabytes of data from the web forums of over 20 different websites.

## 1 Introduction

The extraction of useful information from websites, referred to as scraping [1], is a significant challenging task on several levels due to the large amount of information available on the internet. First, a scraping system must efficiently access web pages by avoiding non-informative data and duplicate pages. Then, only useful data should be detected and extracted. The extracted data should be represented in an exploitable structure to facilitate data analysis. Privacy is another major concern when manipulating web data [2]. Protecting the identity and private life of the user should be taken into consideration [3, 4], particularly in sensitive domains such as health [5] because an increasing number of users today are sharing their personal information on social media such as web forums.

A web forum is a virtual platform for expressing personal and communal opinions, comments, experiences, thoughts, and sentiments [6]. Extracting data from these online communities can produce rich and diverse knowledge resources [7, 8]. The specificity of web forums is that they share a common layout. In particular, the posts are presented in chronological order and organized within threads. This well-organized structure is very useful for targeting specific data within forums. In general, data extraction from web forums involves retrieving the links that lead to threads or posts and obtaining the actual data objects of those threads and posts. A data object can be any information related to user participation in the forum, such as the publication date, author pseudonyms and the post title or content.

In this paper, we present Vigi4Med Scraper, a framework that extracts data objects from web forums and represents them in a semantic structure while maintaining the user’s privacy. The Vigi4Med Scraper framework consists of three main blocks: data extraction from web forums, semantic data representation and anonymization. Whereas each one of these functionalities corresponds to an active research field, we combine them in a highly configurable solution. Our system generates anonymized semantic graphs from any forum-like website according to a user-determined configuration file. With this configuration file, the user can freely specify the desired segments of the forum to extract and denote the correspondence between these segments and the desired semantic components. This flexibility in choosing the segments of data to extract allows Vigi4Med Scraper to handle any forum-like website, which positions it as a generic solution for data extraction from web forums.

Vigi4Med Scraper was used within a pharmacovigilance project. Pharmacovigilance is defined by the World Health Organization as “the science and activities relating to the detection, assessment, understanding and prevention of adverse effects or any other drug-related problem” [9]. In this domain, analyzing web forums is an appropriate way to generate new knowledge about adverse drug reactions (ADRs) [10]. The task imposes two strict requirements for data extraction policy: protecting the privacy of forum users and preserving the performance of the targeted sites. Protecting user privacy is extremely critical when handling personal health data; however, most of the existing web crawling and data extraction approaches blindly gather all types of information without any consideration of privacy. In addition, medical forums are exceedingly popular and have large-scale usage. Thus, the basic requirement of preserving the performance of the crawled websites should be strictly fulfilled, and a particularly respectful attitude towards the hosting server of medical forums should be considered.

This paper is organized as follows. We start by presenting an overview of related work in Section 2. The overall structure of the Vigi4Med Scraper is described in detail in Section 3. Section 4 shows how the framework was applied to the Vigi4Med project. A discussion comparing our system with previous work is proposed in Section 5. Finally, the availability of Vigi4Med Scraper and future directions are presented in Section 6.

## 2 Related Work

Obtaining data objects from web forums involves crawling for informative pages and extracting structured data to precisely retrieve the data of interest within a page. Crawling web forums has been addressed in several studies [11, 12]. The Board Forum approach [13] simulates the natural process of navigating through a forum. It starts by collecting the links from the home page and lower levels (“board”, “thread”) on up to the “post” level. This approach does not extract data objects as it does not process the structure of the collected pages. Later, iRobot was proposed by [14] to crawl web forums. This approach has an offline component that extracts the sitemap (or link skeleton) from sample pages and tries to find the optimal traversal path from one page to another to avoid duplicate pages. The initial version of iRobot did not retrieve specific data objects from web forums, but an extension was proposed in [15, 16]. This new approach also used an offline sampling mode to build the site map; however, it explicitly considered page-flipping links, allowing it to recognize posts belonging to the same thread (or threads belonging to the same forum), even if they were split into several HTML pages. Although [16] also retrieves data objects from web forums, the performance of iRobot depends heavily on the quantity and quality of the sampled pages. Furthermore, iRobot was proven to have ineffective robustness by [17], who proposed a new approach called FoCUS (Forum Crawler Under Supervision). FoCUS [17] learns regular expression patterns to extract the main features from a sample collection and uses these expressions to direct online crawling. The authors evaluated their approach for high scale crawling. A selected number of data objects were used as features for a Support Vector Machine (SVM) classifier. The data objects were chosen to help the classifier distinguish a board page from a thread page, but the extraction of these data objects was not the final goal of the approach.

Approaches that extract structured data from web pages have been extensively studied. The procedures implemented to achieve structured data extraction are called wrappers [18]. Several techniques, such as regular expressions and tree-based methods, can be used to generate a wrapper. The Document Object Model (DOM) is commonly used to extract data from web pages. A DOM tree represents the pages’ information in a structure that can be exploited by special queries (XPath queries). Although manual approaches allow one to specify the data of interest, they rely heavily on users with the appropriate technical expertise. Automatic approaches were introduced to lower the amount of user effort required for this task. The majority of these approaches still require human intervention to label training examples (e.g., [19]). Fully automatic approaches try to detect nested or repeated patterns to target interesting contents (e.g., [20]), but they suffer from a higher risk of extracting non-informative data and are difficult to customize.

None of the previous studies focused on the semantic representation of data or privacy. Semantic representation allows for a powerful and flexible description of knowledge. Quickly after the emergence of the Semantic Web [21], this type of representation, which is based on concepts and semantic relations, has garnered important interest, particularly in the medical domain [22]. Privacy is a main concern in web forum crawling. Protecting the privacy of web-collected data is a complicated issue [23]. Any information that can identify a specific user should not be straightforward to reveal, particularly when working with health data within a medical domain such as pharmacovigilance. One way to protect privacy is to anonymize sensitive data. In the literature, in addition to basic pseudonymization (replacing an identifier with a key), several approaches exist for anonymization, such as k-anonymat [24, 25] and differential privacy [26]. The choice of an anonymization algorithm depends on the context of the application. In particular, it depends on who has access to which part of the data, and whether the anonymization is desired to be reversible or not [27].

With respect to previous research, each of the aforementioned web forum data extraction approaches lacks at least one of the following elements:

- Efficiency: Avoiding network overload by ignoring duplicate pages and non-informative data;
- Page flipping: Maintaining the logical connection between posts belonging to the same thread presented over several pages;
- Data Object Detection: Detecting precise information related to posts, threads and authors;
- Conceptual representation: Using semantic graphs to store extracted data;
- Privacy: Protecting personal data;
- Availability: Providing publicly available implementation and documentation.

Our framework was designed to address these issues, as we describe in the following sections.

## 3 Framework Description

**Fig 1:**
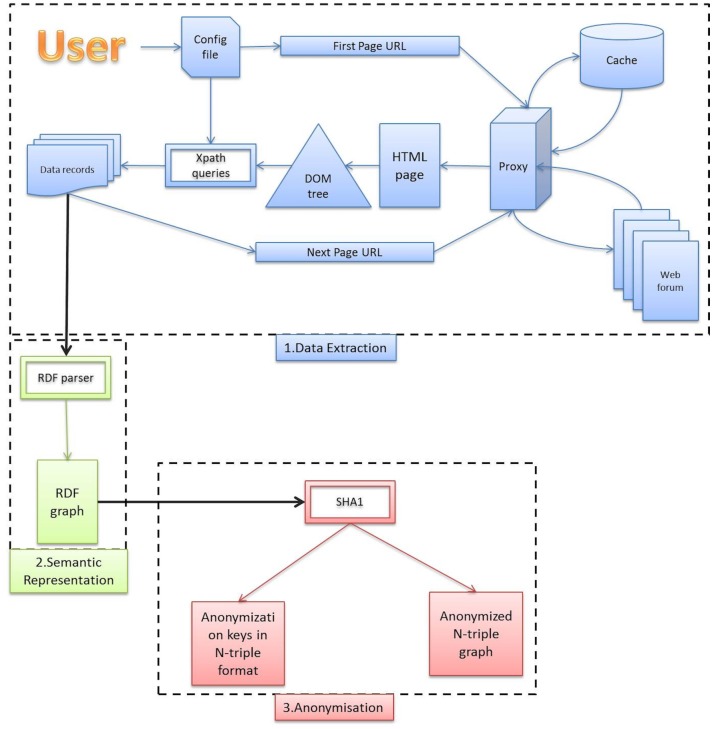
Vigi4Med Scraper Structure.

As mentioned earlier, Vigi4Med Scraper consists of three main functionalities (Fig 1): data extraction from web forums, semantic data representation and anonymization. Each of these functionalities is described in detail in the following subsections.

### 3.1 Data Extraction

**Fig 2:**
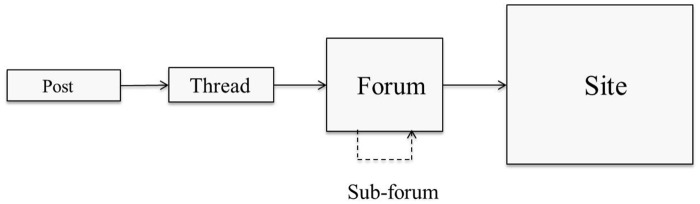
Web forums structure.

**Fig 3:**
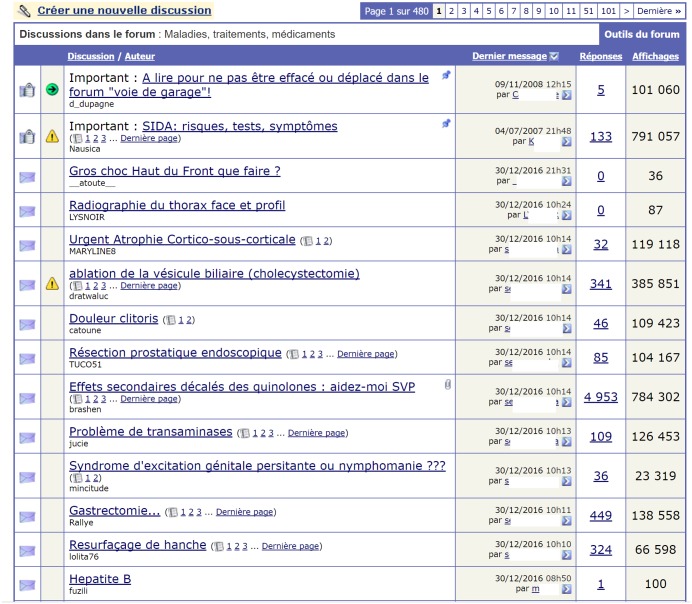
An example of threads within a forum page.

**Fig 4:**
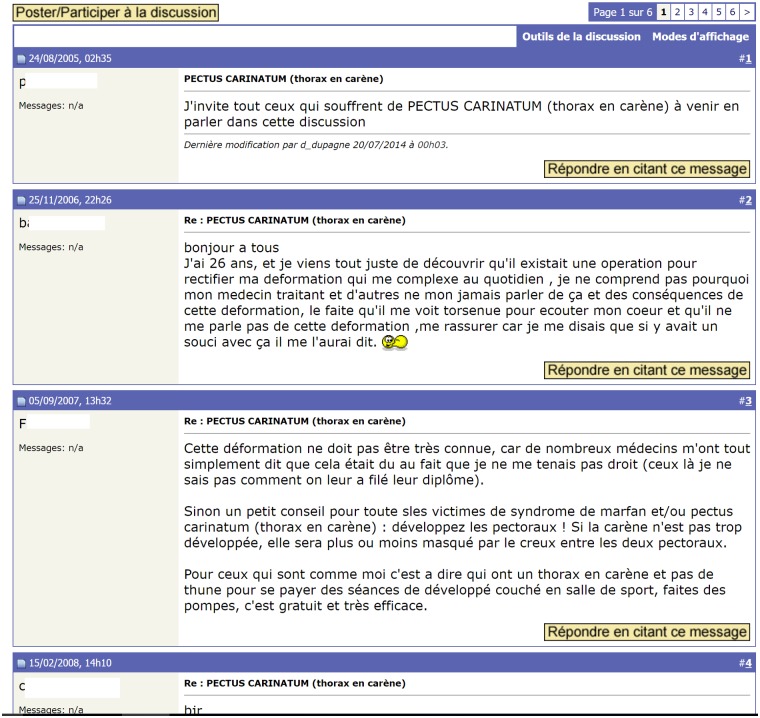
An example of messages within a thread page.

We adopted a scraping approach that simulates the spontaneous behavior of a user exploring a web forum. This behavior is based on the general structure of a web forum (Fig 2), where each post belongs to a thread, and each thread belongs to a forum. Additionally, a website can contain several web forums occasionally organized into a hierarchy. To navigate through a web forum, a user typically starts at the board page, targets a thread, and explores the posts in the thread. If the posts (or threads) are split across multiple pages, the “next page” link is used to access subsequent pages. To automatize this user behavior, our algorithm attaches the identifier of each forum or thread to all its related elements, even if they appear in several pages, as we will see in Section 3.2. In the example of Fig 3, we see a forum page with a list of threads. Each thread in this page is associated with several data objects, such as the link to the posts in the thread (which is also the title of the thread), the number of replies (*Réponses* in French) and the number of views (*Affichages* in French). Clicking a thread link leads to a thread page. The thread page (Fig 4) contains a list of posts, and each post has several data objects. Examples of post data objects include the post content, author name and publication date. In Figs 3 and 4, navigating to the next page, whether for threads or posts, is possible using the symbol “>”.

The scraping function uses dynamic lists of URLs for extracting data. This list is initialized with a file containing user-provided forum URLs that is automatically expanded with the URLs of the threads scraped from these forums. Each scraped URL is removed from the corresponding list for tracking in the event of unexpected corruption. Simultaneously, each scraped URL is added to a log file, which is checked each time a new URL is requested. This allows the algorithm to ignore duplicate requests. After an HTML page is retrieved, the algorithm generates a DOM (Document Object Model) tree. In this tree, each data object is a node that can be retrieved using Xpath queries [28]. If an Xpath query describes a non-unique node, it retrieves a list of all the objects that satisfy that Xpath. This behavior is used in our framework to efficiently extract similar objects that share the same XPath prefix. In web forums, these objects are related to posts in a thread page or threads in a forum page (cf. Figs 3 and 4). Because the addition of these objects to an HTML page on the server side is automatic, they inevitably belong to the same DOM parent node and share several exclusively identifiable characteristics. For example, the post authors in a thread page all belong to a “td” element within a table called “posts-table”, and they all have an identifier that ends with the string “-postAuthor”. The advantage of using Xpath queries is that it avoids the noise generated by ads and non-informative data, which generally do not follow the same pattern as the extractable objects that are of interest to the user. To specify the Xpaths that the algorithm must use, the user manually fills out a configuration file (cf. Section 3.1.1) with the description of the desired objects for each scraped website.

#### 3.1.1 Configuration File

Vigi4Med Scraper requires a configuration file that contains the Xpaths of the objects the user wants to extract from the forums of a website. As shown in the Listing example 1, these Xpaths are organized into two sections, one for threads “Threads-Info” and the other for posts “Messages-Info”. The number and the nature of the objects to be extracted are not pre-defined. The only constraint is the specification of the elements “sioc:Thread” and “sioc:Post” (details in section 3.2), which contain the Xpath of the identifier of a thread or a post, respectively, and the element “nextPage” (for threads and posts) to provide the Xpath of the next page’s URL. Apart from these particular elements, the algorithm will take into account any object described in the configuration file as long as it matches a pattern that the algorithm can recognize. We describe this pattern later in Listing 4. In addition to these two sections, the configuration file contains proxy information and a regular expression that describes the format of the “date” objects (e.g., post publication date) in the scraped website. This regular expression is optional; it is used by the algorithm to standardize the date representations in the output RDF [29] file. The section “Files info” contains the input and output file names. The input file is the list of forums’ URLS to scrape, and the output files are the log and the generated RDF graph.

**Listing 1. Configuration file example**

proxy = ‘’

dateFormat = ‘* * d-m-Y*?? H: i: s??’

[Files_Info]

forumsInputList = ‘configFiles/ForumsLists/Doctissimo_Forums.txt’

logFileName = ‘../logs/Doctissimo_’’

rdfFileName = ‘../download/Doctissimo/Doctissimo_Graph. n3’

[Threads_Info]

sioc: Thread = ‘//*[starts-with(@id, ’url_topic_’)]’:: id

dc: creator = ‘//*[@id = ’block_topics_list’]/ tr/ td [6]’

dc: title = ‘//*[starts-with(@id, ’url_topic_’)]’

sioc: num_replies = ‘//*[@id = ’block_topics_list’]/ tr/ td [7]’:: xsd: integer

sioc: num_views = ‘//*[@id = ’block_topics_list’]/ tr/ td [8]’:: xsd: integer

nextPage = ‘//*[@id = ’block_topics_list’]/ tr [2]/ td / div [1]/ div [1]/ a’

[Messages_Info]

sioc: Post = ‘//td[@class = ’messCase1’]/ div [1]/ a[1]/@href’:: id

dc: creator = ‘//td[@class = ’messCase1’]/ div [2]’

dc: date = ‘//td[@class = ’messCase2’]/ div [1]/ div [1]’:: xsd: dateTime

sioc: content = ‘//*[starts-with(@id, ’para’)]’:: fr

nie: htmlContent = ‘//*[starts-with(@id, ’para’)]’:: rdf: HTML

nextPage = ‘//*[@id = ’topic’]/ table [1]/ tr [2]/ td/ div [1]/ div [1]/ a [1]’

#### 3.1.2 Proxy and Cache

Scraping web forums implies a significant number of connections to the websites from which we want to extract data. In order to minimize the network load and avoid overwhelming the destination servers, it is important to avoid duplicate requests and have a delay between sequential connections. In web forums, a web page can be scraped several times if a thread belongs to several sub forums or if the scraping process is restarted for any reason. To handle the first case, a log file keeps track of all the visited URLs in a website. Before requesting a new URL, the scraping script checks if the URL has already been visited, in which case, it will be ignored. To handle unexpected issues relating to retrieving a URL that was already visited, a proxy with a cache database is proposed. Before opening a new connection with the destination server, the scraping function checks if its database already contains the desired HTML page. If so, the HTML page is sent back to the scraping function; otherwise, a connection is established with the destination server, and a copy of the received HTML page is saved to the cache. Moreover, the proxy maintains a minimum delay of 0.330 seconds between two sequential requests. This delay is extended to 2 seconds during working hours at our university to preserve the performance of the network.

#### 3.1.3 Data Extraction Summary

As we see from Fig 1, the data extraction step can be summarized as follows. The user fills out one configuration file per site. In this file, she provides the list of forums that she wants to scrape. For each URL on this list, the scraping function generates another list that contains the URLs of all the available threads in the current forum. This list is then used to scrape the posts in each thread prior to scraping the threads of the subsequent forum. The scraping function is identical for both threads and posts, and the configuration file tells the function which objects to extract in each case. Before requesting an URL, the scraping function starts by checking the logs. If the URL was previously scraped, it will be ignored; otherwise, it is sent to the proxy. The proxy will either retrieve the corresponding HTML page from its cache or fetch the page from the destination website. When the HTML page is ready, a DOM tree is generated, and the Xpath queries in the configuration file are executed against the DOM tree to obtain structured data records. For example, if the user defined the Xpaths of the posts’ titles, creators and publication dates in the configuration file, the algorithm will produce structured data records with the values of these fields for each post in each scraped HTML page. Vigi4Med Scraper will keep navigating (in thread or post pages) until no match is retrieved for the Xpath defined in the element “nextPage”. This naturally happens when we reach the last page of threads (or posts), where the link that leads to the next page is absent.

### 3.2 Semantic Data Representation

In order to represent the collected data in a flexible and efficient structure, we use a RDF graph with N-triples syntax [28]. Each line in the generated N-triples file is a sequence of subject, predicate and object separated by whitespace and terminated with a “.” after each triple. Vigi4Med Scraper does not presume a unique inner-structure for threads and posts, as these data can differ from one site to another and depend on the requirements defined by the user for a specific task. Nevertheless, the system is designed to scrape web forums. Three elements are thus mandatory: navigation through pages for both threads and posts, the identification of a thread, and the identification of a post. The special element “nextPage” should be used to declare the XPath corresponding to the “nextpage” navigation link; the extracted link will only be used for crawling and will not appear in the resulting RDF graph. The elements “sioc:Thread” and “sioc:Post” are used to declare the Xpaths to the URLs of the threads and the posts as shown in the examples presented in Listing 2 and Listing 3, respectively.

**Listing 2. Thread identifier definition in the configuration file**

sioc: Thread = ‘//*[starts-with(@id, ‘url_topic_’)]’:: id

**Listing 3. Thread identifier definition in the configuration file**

sioc: Post = ‘//td[@class = ‘messCase1’]/ div[1]/ a[1]/ @href’:: id

These elements generate triples in the RDF graph, where the subject is the extracted URL, which is used as an identifier, the predicate is the RDF relation “type”, and the value is the corresponding semantic vocabulary describing a thread or a post. The generated identifiers are also used as subjects for the semantic attributes defined by the user in the remaining thread and post sections. To define a semantic attribute, the user states the desired predicate as well as the Xpath that leads to its value for threads or posts. This is achieved by using the pattern described in Listing 4.

**Listing 4. Pattern accepted in the configuration file**

RDF_Predicate_name = XPath_address:: Rdftype

**Fig 5:**
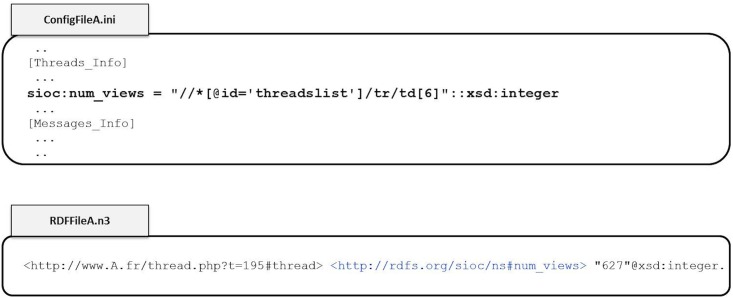
Semantic data representation.

In this pattern, the name of the predicate generated in the N-triple file is specified by “RDF-Predicate-name”. The Xpath that leads to the desired data value is “XPath-address”, which corresponds to the object of the triple. The user is free to specify the semantic vocabulary used to define the predicates. The most commonly adopted standard to describe forums elements is SIOC [30]. For example, the property “sioc:num_replies” defines the number of replies within a thread. Other vocabularies can be used to describe generic (not forum specific) properties, such as “dc:date” from Dublin Core [31], which has broad and generic elements to describe a wide range of resources, or “nie:htmlContent” from NEPOMUK Information Element Ontology [32], which describes native resources available on the desktop. “RDFtype” is optional; if it is declared, the type will be added to the value of the generated triple. In the example of Fig 5, the Xpath leading to the number of thread views is specified in the configuration file “ConfigFileA”. The pattern in this example specifies that this object corresponds to the semantic predicate “sioc:num_views” of type “integer”. Using this configuration, the corresponding triple is generated in the output file “Temp-FileA.n3”.

**Fig 6:**
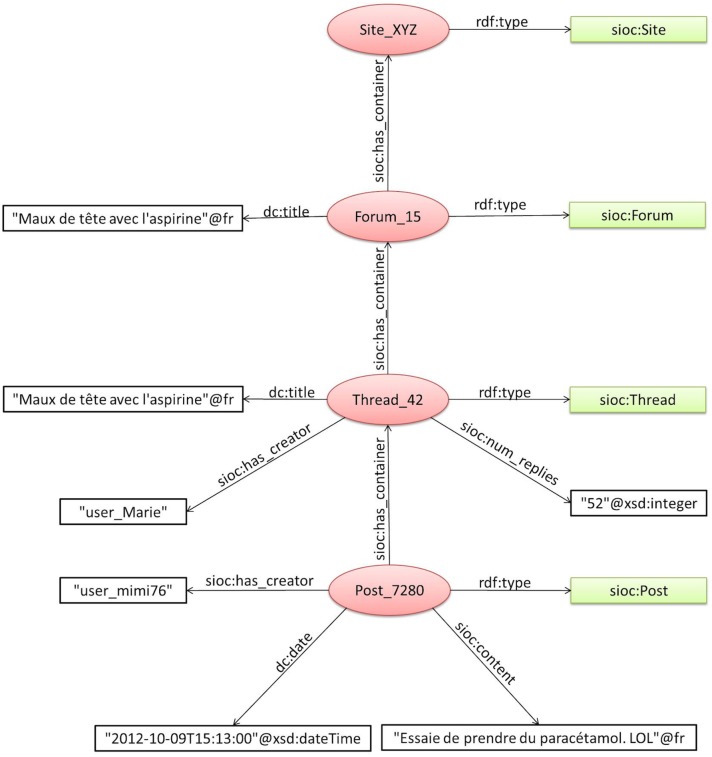
An example of the generated RDF graph.

To demonstrate RDF graph generation, Fig 6 shows an example of a generated sub graph for a post. In this example, the post “Post_7280” has the type “sioc:Post, and it is associated with the author, text and date by the semantic relations “sioc:creator”’, “sioc:content”, and “dc:date”, respectively. The “Post_7280” belongs to the thread “Thread_42” (of type sioc:Thread), which is associated with the title, creator, and the number of replies via the semantic relations “dc:title”’, “sioc:creator”, “sioc:num_replies”, respectively. Finally, this thread appears in the forum “Forum_15” (of type sioc:Forum), which belongs to the website “Site_XYZ” (of type sioc:Site).

### 3.3 Anonymization

**Fig 7:**
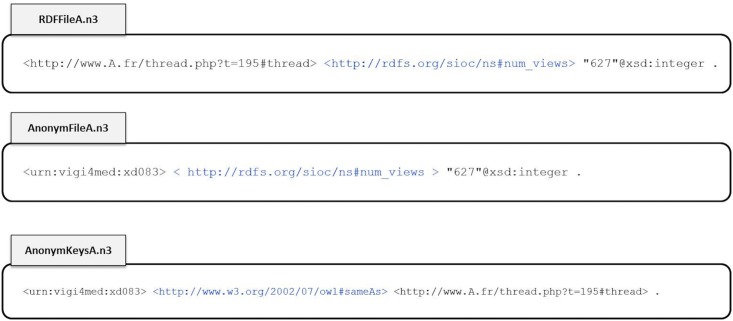
Anonymization.

Pseudonymization is applied in the current version of the framework. We defined authors’ pseudonyms, profile information, and the URLs of posts and threads as the identifiers to anonymize. Each identifier is replaced by a key generated by the cryptographic hash function SHA1 (Secure Hash Algorithm 1). The semantic representation is preserved for both the anonymized data and the anonymization keys. In other words, we generate two N-triple graphs, one for the anonymized objects and the other for the anonymization keys. For example, in Fig 7, the file “RDFFileA.n3” has a triple regarding the number of views of a thread. The identifier of this thread (its URL) is anonymized in the file “AnonymFileA.n3”, while the anonymization key is kept in another file “AnonymKeysA.n3”.

It is important to note that to break the anonymization, we can simply concatenate both the anonymized data and the corresponding anonymization key files. Recognizing this concatenation, an RDF parser can find the connection between the anonymized triples and their keys in the same file. One may argue that anonymization should be irreversible. However, we hypothesized that the retrieval of original identifiers should be allowed in specific cases. For example, in pharmacovigilance, the detection of a dangerous case of drug exposure might necessitate notifying the patient to contact her physician. Nevertheless, our framework is designed to generate a separate graph of anonymization keys, which should be kept in a safe place during normal usage. In addition, these keys should not be used by the team that processes the anonymized data.

In the example of Fig 6, the anonymization process generates a new graph where the nodes “post_7280”, “Thread_42”, “Forum_15”, “Site_XYZ”, “user_Marie” and “user_mimi76” are replaced by the anonymization keys obtained by SHA1.

To guarantee the validity and the quality of our RDF graph, the anonymization process distinguishes three author profiles: known authors (users with a profile page), unknown authors (nicknames with no corresponding profile page) and invalid authors (anonymous users or deleted profiles). Each valid author has three corresponding triples in the RDF graph (ex. Listing 5): the first triple describes his type (Person), the second identifies his profile page, and the third links to his nickname. Thus, to anonymize the user’s information, the script will anonymize the author’s nickname and profile page for known authors. Only the nicknames of unknown authors are anonymized, and a blank node [33] is created to represent the missing profile pages of these authors. Invalid authors do not appear in the graph, and their corresponding posts ae considered to be posts with no authors (i.e., these posts will not have the semantic property “dc:creator”). The choice to ignore invalid authors is important for subsequent data analysis procedures because although posts written by deleted or anonymous authors typically have one unique string for the author’s nickname, i.e., “Unknown” or “Deleted profile”, this does not indicate that these posts have been written by the same user. The anonymization script is parametrized to take the strings that distinguish invalid authors in the forums as input and automatically detect the users with no scraped profile information to generate the corresponding blank nodes. In Listing 5, we show the anonymization results of an unknown user profile with the nickname “XYZ”. Only the first triple will be present in the anonymized graph, while the remainder are kept in the anonymization key file.

**Listing 5. Anonymizing unknown users**

<urn: vigi4med: 0001021 f68> <http://www.w3.org/1999/02/22-rdf-syntax-ns#type>

   <http://xmlns.com/foaf/0.1/Person>.

<urn: vigi4med: 0001021 f68> <http://www.w3.org/2002/07/owl#sameAs>

   <_: id878140c00e295edea3a81d00>.

<urn: vigi4med: 0001021 f68> <http://xmlns.com/foaf/0.1/nick>

   “XYZ”.

## 4 Application to the Vigi4Med Project

Adverse drug reactions are often mentioned in medical-related discussions. The extraction of knowledge from web forums has recently received attention in the scientific community to exploit this complementary data source. Indeed, clinical trials are essential for identifying ADRs; however, they are expensive and time consuming and cannot detect all possible reactions, particularly uncommon reactions. This is because a limited number of patients are enrolled in these trials, and children and pregnant women are often not considered in them. Moreover, during the post marketing phase, patients and health professionals do not report every ADR to safety agencies or the pharmaceutical industry, even when such reporting is mandatory for health professionals. ADRs are a common cause of morbidity. For example, in France, the estimated annual number of ADR-related hospitalizations is greater than 140 000 [34], which explains why new strategies to address the problem of under-reporting are being embraced. Patient feedback through online social networks is a non-negligible resource for potentially reducing the number of deaths and hospitalizations due to adverse drug reactions [35]. The efficient detection of ADRs from such resources could finalize and/or confirm the results of clinical trials and post-marketing reports. It also allows for the expedited detection of potential ADR signals that have gone unnoticed in previous sources that might emerge at a later time. In this context, the French drug safety agency ANSM (French acronym of *Agence Nationale de Sécurité du Médicament et des produits de santé*) founded the Vigi4Med project in 2013 [4]. The main goal of ANSM is to detect ADRs from posts in social networks, particularly medical-related web forums. To achieve this goal, the partners of the Vigi4Med project have agreed upon the following protocol:

1. A declaration about extracting medical information from web forums is sent to the CNIL [36], the national organization of data protection in France.
2. Pharmacovigilance experts select the websites with medical-related forums.
3. A message is sent to the forum’s owners and administrators to inform them about the motivation of the project and their data crawling policy. This message also verifies our commitment to refrain from distributing or republishing the collected data.
4. The scraping algorithm respects a pre-defined delay between requests.
5. The scraped data are anonymized and organized in a semantic structure before proceeding with any further steps.

The project involves several partners; each partner is responsible for one specific task in the project. Vigi4Med Scraper was designed to complete the tasks of data extraction, semantic representation and anonymization. The anonymized graph generated by the first partner using the framework is further processed by another project partner to realize the annotation task. The pharmacovigilance end users are represented by two regional centers acting as partners in the project. These users are in charge of comparing case reports in the French spontaneous reporting system with potential ADRs identified within patients’ posts after the annotation step.

**Fig 8:**
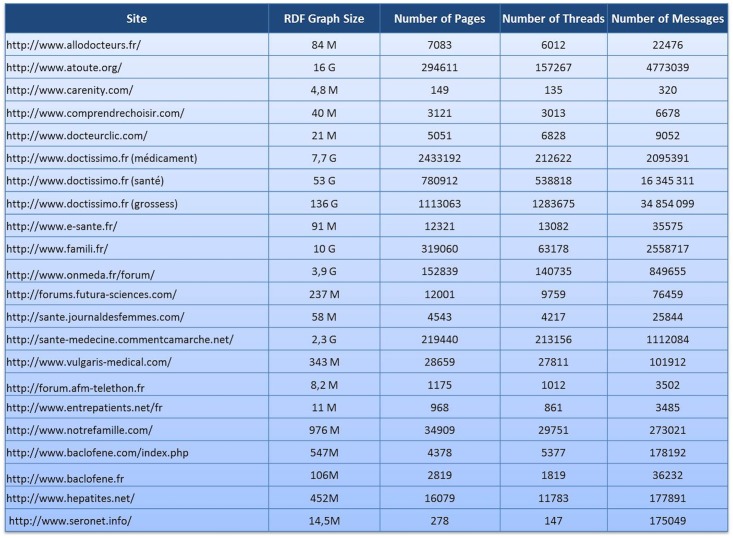
Scraped sites results.

The application of our framework in this project involved an auxiliary script to extract a list of forums for each site chosen by the pharmacovigilance experts. This list was filtered manually to exclude non medical-related forums. For example, the forum “fashion” was eliminated from the forum list of the website “www.doctissimo.fr”. This list was the starting point of the extraction process, along with its parameterization in the Vigi4Med Scraper configuration file. Because we hypothesized that a non-related medical discussion within a medical forum could indirectly lead to information about adverse drug reactions, no specific selection was made to filter the threads and posts. In addition, the extracted content was annotated (by the following partner) to discard irrelevant posts. Regarding anonymization, we considered that pseudonymization was suitable for this project as none of the partners has access to both the anonymized data and the anonymization keys. As a result, 55 websites were selected by the pharmacovigilance experts. Among these websites, 22 were scraped between January and June 2015. The scraping within this period was not continuous, and it always respected the specified delays between sequential requests. Over 60 million posts, 2.5 million threads, and 5.4 million pages corresponding to more than 200 gigabytes of data were collected. Fig 8 shows the size of the generated RDF graph, the number of pages, and the number of threads and posts of each scraped website in this experiment. The privacy protocol was strictly followed. None of the involved website owners objected to the crawling process. The anonymized semantic graphs were delivered to the Vigi4Med partner in charge of ADR annotation.

## 5 Discussion

Vigi4Med Scraper offers a freely available open source framework to retrieve data objects from web forums. Vigi4Med Scraper employs a forum crawling strategy based on natural navigation and a data extraction approach based on DOM structure. The framework is highly configurable and can be adapted to any forum-like website. Privacy is handled by explicitly anonymizing any data objects that can potentially reveal a person’s identity. The semantic representation in an RDF graph offers a harmonized structure that allows for straightforward manipulation by data analysis algorithms. With this representation, integrating the collected data with an existing semantic resource can be directly achieved. In other words, the resulted RDF graph follows the standard syntax and serialization format defined by the World Wide Web Consortium (W3C); thus, it is straightforward to link it to other existing RDF graphs or extend it with new concepts and semantic relations. The valid conceptual representation in Vigi4Med Scraper acknowledges the nature of forums, as the organizational structure of the forums and the page flipping aspect are naturally represented in the RDF graph. Furthermore, Vigi4Med Scraper is extremely selective; it will not blindly explore all the available links and data in a page but instead utilizes the specific “next page” link, which also allows it to maintain the logical connection between posts (or threads) across several pages. This selective behavior has the advantage of avoiding non-informative data. For example, advertising posts, which generally do not have the same structural characteristics as normal posts, are invisible to our algorithm. Duplicate pages will only be scraped once because our solution keeps track of previously accessed links, and the proxy ensures that no additional requests are sent to previously accessed pages. The proxy also guarantees a minimal delay between sequential requests to avoid network and server overload. Although the requirement of having a trained user fill out the configuration file can be considered as a potential limitation of the framework, it guarantees accurate and efficient data extraction. In addition, such user intervention can be facilitated by the DOM inspection tools of several internet browsers. For all these reasons, Vigi4Med Scraper was the adopted solution for extracting posts from several medical-related forums within the Vigi4Med project.

The objective of our work focuses on the quality and the usability of the results, which cannot be quantified by experimental measures. Moreover, because we voluntarily added a delay between successive requests to prevent network overload, considering the execution time as a quantitative measure does not apply to our case. Thus, to compare our solution with those of previous researchers, we considered the six essential criteria described in Section 2: efficiency, page flipping consideration, data object detection, conceptual representation, privacy and availability. As we summarized in Section 2, none of the existing systems meets all these requirements. Table 1 shows a direct comparison of our system with other systems on these criteria. In this table, the symbol “✕” denotes that the criteria is not relevant or it is not handled explicitly by the studied approach. With this consideration, our defined efficiency criteria does not concern data extraction approaches [19, 20] as they do not treat problems related to accessing web pages and network overload. Thus, they are not concerned about data objects related to the navigation, like page flipping links. Although all crawling approaches are designed to maximize efficiency [13–17], only some of them [15–17] consider the page flipping issue, which is critical for web forum crawling. With the exception of the work of [17], these approaches do not focus on extracting data objects from crawled web pages. Unlike Vigi4Med Scrapper, none of the aforementioned approaches addresses semantic representation or privacy. In addition, the implementations of these approaches are not publicly available.

**Table 1:** Comparaison of Vigi4Med Scrapper and other similar systems.

Approach	Efficiency	P.flipping	Data ObDet.	Concept. Rep.	Privacy	Availability
Muslea et al. [19]	✕	✕	✔	✕	✕	✕
Crescenzi et al. [20]	✕	✕	✔	✕	✕	✕
Guo et al. [13]	✔	✕	✕	✕	✕	✕
Cai et al. [14]	✔	✕	✕	✕	✕	✕
Wang et al. [15]	✔	✔	✕	✕	✕	✕
Yang et al. [16]	✔	✔	✔	✕	✕	✕
Jiang et al. [17]	✔	✔	✔	✕	✕	✕
Vigi4Med Scrapper	✔	✔	✔	✔	✔	✔

## 6 Availability and Future Directions

We have presented Vigi4Med Scraper, a generic tool to extract structured information from web forums. Vigi4Med Scraper is part of the Vigi4Med project for detecting adverse drug reactions in social networks. All the scraping and anonymization scripts were implemented in PHP. The proxy is a PERL program that is connected to a database (berkeleyDB [19]) for caching. To run the system, a PHP server and PERL installation are required. The complete source code is verbosely commented and publicly available under the *GNU* open source license. It can be accessed at the following address: https://github.com/bissana/Vigi4Med-Scraper. Full documentation (in English and French) regarding the code and configuration file is also provided at this URL.

Although the configuration file for Vigi4Med Scraper guarantees the maximum flexibility of the application, preparing such a file is a sensitive step requiring special attention. A complementary tool that helps the expert initialise the configuration file would be helpful for the preparation phase. In addition, a user-friendly interface that controls the grammar of the free parameters and proposes default configuration settings would prevent errors and increase efficiency. Because the framework does not currently handle client-side generated scripts, authenticated access, or encrypted pages, adding a specific module to handle these cases would be an interesting extension of our work. Finally, the maintenance of DOM-based approaches is a critical issue in the literature of web data extraction because the structure of online pages is unstable and can be modified repeatedly. Although this was not a problem for the project (which was a one-shot process to extract retrospective data), analyzing the state of the art on this issue would help us gain an important perspective for improving our framework.
